# Multimodality Characterization of Cancer-Associated Fibroblasts in Tumor Microenvironment and Its Correlation With Ultrasound Shear Wave-Measured Tissue Stiffness in Localized Prostate Cancer

**DOI:** 10.3389/fonc.2022.822476

**Published:** 2022-04-21

**Authors:** Wael Ageeli, Xinyu Zhang, Chidozie N. Ogbonnaya, Susan E. Bray, Neil M. Kernohan, Jennifer Wilson, Chunhui Li, Ghulam Nabi

**Affiliations:** ^1^ Division of Imaging Sciences and Technology, School of Medicine, University of Dundee, Ninewells Hospital, Dundee, United Kingdom; ^2^ Diagnostic Radiology Department, College of Applied Medical Sciences, Jazan University, Jazan, Saudi Arabia; ^3^ Division of Population Health and Genomics, School of Medicine, University of Dundee, Dundee, United Kingdom; ^4^ Tayside Biorepository, Ninewells Hospital & Medical School, University of Dundee, Dundee, United Kingdom; ^5^ Department of Pathology, Ninewells Hospital, Dundee, United Kingdom; ^6^ School of Science and Engineering, University of Dundee, Dundee, United Kingdom

**Keywords:** cancer-associated fibroblasts, prostate cancer, immunohistochemistry, stiffness, tumor microenvironment

## Abstract

**Introduction:**

Growing evidence suggests that the tumor microenvironment (TME) represented by cellular and acellular components plays a key role in the multistep process of metastases and response to therapies. However, imaging and molecular characterization of the TME in prostate cancer (PCa) and its role in predicting aggressive tumor behavior and disease progression is largely unexplored. The study explores the PCa TME through the characterization of cancer-associated fibroblasts (CAFs) using both immunohistochemistry (IHC) and genomics approaches. This is then correlated with transrectal ultrasound shear wave elastography (USWE)-measured tissue stiffness.

**Patients and Methods:**

Thirty patients with clinically localized PCa undergoing radical prostatectomy for different risk categories of tumor (low, intermediate, and high) defined by Gleason score (GS) were prospectively recruited into this study. Prostatic tissue stiffness was measured using USWE prior to surgery. The CAFs within the TME were identified by IHC using a panel of six antibodies (FAP, SMAα, FSP1, CD36, PDGFRα, and PDGFRβ) as well as gene expression profiling using TempO-sequence analysis. Whether the pattern and degree of immunohistochemical positivity (measured by Quick score method) and expression of genes characterizing CAFs were correlated with USWE- and GS-measured tissue stiffnesses were tested using Spearman’s rank correlation and Pearson correlation.

**Results:**

There was a statistically significant correlation between GS of cancers, the pattern of staining for CAFs by immunohistochemical staining, and tissue stiffness measured in kPa using USWE (*p* < 0.001). Significant differences were also observed in immunohistochemical staining patterns between normal prostate and prostatic cancerous tissue. PDGFRβ and SMAα immunostaining scores increased linearly with increasing the USWE stiffness and the GS of PCa. There was a significant positive correlation between increasing tissue stiffness in tumor stroma and SMAα and PDGFRβ gene expression in the fibromuscular stroma (*p* < 0.001).

**Conclusion:**

USWE-measured tissue stiffness correlates with increased SMAα and PDGFRβ expressing CAFs and PCa GSs. This mechanistic correlation could be used for predicting the upgrading of GS from biopsies to radical surgery and response to novel treatments.

## Key points:

1. There is heterogeneity in the amount and types of CAFs in the TME of localized PCa and this correlates with tissue stiffness (elastography) on imaging.
2. Immunohistochemical expression of SMAα by CAFs also correlates with GS of PCa and was significantly different from normal benign prostate tissue.
3. USWE-measured tissue stiffness imaging provides an insight into TME and the presence of CAFs in localized PCa.

## 1 Introduction

Several studies indicate that the biophysical and biochemical features of the connective tissue stroma within the normal prostate are different from those of the tumor and that the properties of the tumor stromal elements have a significant impact on cancer development and progression (1–4). Similar to other cancers, prostate cancer (PCa) is not now seen simply as a disease of abnormally proliferating epithelial cells, but rather as diverse diseases arising from the complex interactions between the cells of the prostatic epithelial compartment and the surrounding stromal environment in which they sit (5). PCa has a high degree of heterogeneity in tumor genotype, phenotype, and behavior (including responsiveness to therapy) between tumors of the same histological type in different patients.

There is a cross-talk between cell types within the tumor microenvironment (TME) where one of the most important and abundant cell types is the CAFs (5). CAFs form the largest population of stromal cells in TME (6) and have been found to induce an alteration in the architecture and physical properties of the ECM that promote cell migration, invasion, and tumor growth (7). Vimentin and FSP1 are expressed by normal fibroblasts embedded in the fibrillar ECM of connective tissue (8) but CAFs can be identified by altered or overexpression of a number of molecular markers that are upregulated compared to normal fibroblasts, including α-smooth muscle actin (SMAα), fibroblast activation protein (FAP), fibroblast specific protein-1 (FSP-1), platelet-derived growth factor receptor-α (PDGFR-α), and platelet-derived growth factor receptor-β (PDGFR-β) (9, 10). Several studies have noted specifically that CAFs can be distinguished by their expression of SMAα (11–13). FAP has also emerged as a potential candidate for directly targeting CAFs (14) as it may be expressed by activated fibroblasts within tumor stroma and healing wound tissue. It is not expressed by mature somatic tissue fibroblasts (15).

The activity of the CAF remodeling is associated with increased tissue stiffness and altered mechanical stress responses (16); several studies discovered that the mechanical characteristics of prostate tissue revealed an increased stiffness within the cancerous stroma compared to the normal stroma (17–19). Our group and others have reported that USWE characterization of tissue stiffness of PCa can be considered as a promising imaging method or technique; previous studies have shown that this parameter correlated with the GS, identifying clinically significant PCa (20–25) with diagnostic accuracy, evident by reported sensitivity, specificity, positive predictive value, and negative predictive values (23, 26, 27).

In the present study, we explored the correlation between CAF heterogeneity within TME and transrectal USWE-measured tissue stiffness. CAF heterogeneity was explored using immunohistochemistry and gene expression. The specific selection of these two methods was based on the understanding that these are complementary technology and would provide reasonable assurance and validity to study findings. Gene expression technique used TempO-Seq assays specifically designed for the study of RNA expression in a formalin-fixed tissue specimen. Knowledge gained through this study could establish or otherwise invalidate the role of non-invasive imaging as a marker of changes in TME and potentially improve prediction of upgrading from biopsy to radical surgery and also facilitate risk stratification of patients with localized PCa. Moreover, differences in the TME (including immune cell infiltration, CAFs, the density of the extracellular matrix, hypoxia, and interstitial fluid pressure) can explain why tumors behave differently to the same treatment modalities.

## 2 Patients and Methods

### 2.1 Study Design and Patients

This was a prospective, protocol-driven study with ethical approval through East of Scotland Ethical committee, approval from the Tayside Tissue Bank, University of Dundee, Ninewells Hospital and Medical School (Dundee, UK) to undertake investigations on patient specimens, and Caldicott permission (IGTCAL5626) to access patient follow-up data.

**Figure 1:**
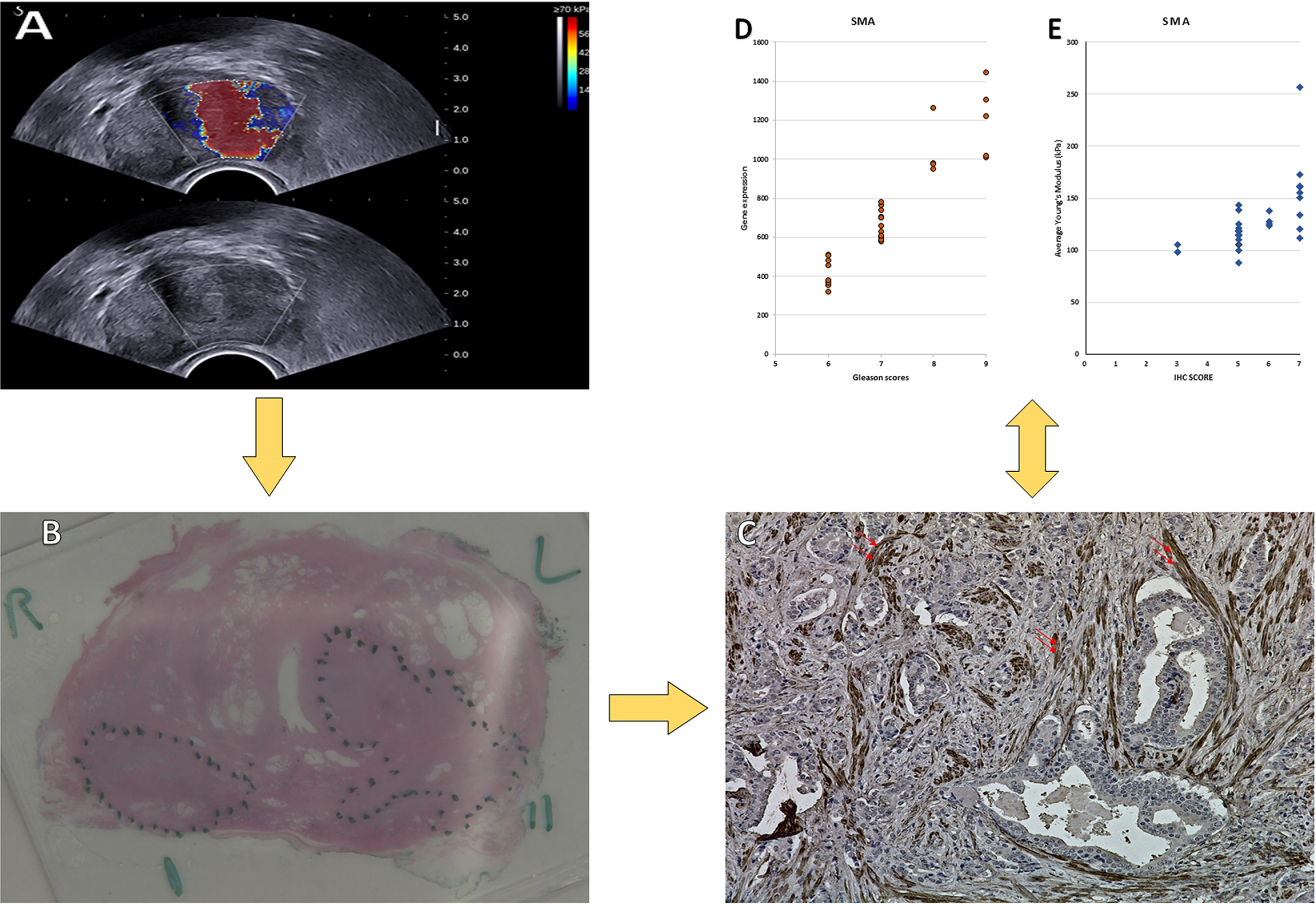
Images obtained from a 72-year-old man. **(A)** USWE image; the average mean of the stiffness was 143.6 kPa. **(B)** Histopathology image of the prostate cancer; the final diagnosis was prostate cancer with Gleason score 4 + 5. **(C)** IHC of SMAα with dilution (1/50 + linker) in prostate cancer tissue. 400× magnification. **(D)** The relationship between the Gleason score and the gene expression of the SMAα. **(E)** The relationship between the Gleason score and the IHC of the SMAα.

Consecutive men with localized PCa undergoing radical surgery were recruited. Pre-operative elastograms of prostate glands were obtained using transrectal USWE. Prostatic tissue stiffness in kilopascals (kPa) prior to radical surgery was measured in all the participants (**Figure 1**). Patients with prior prostatic surgery or radiotherapy or in whom there had been previous rectal surgery precluding placement of transrectal ultrasound examination were excluded. Patient-specific customized 3D molds were printed using imaging and 3-D printers in all the cases according to our published protocol (28).

The histopathology of the cancers within the radical surgery specimens was assessed and graded by an experienced uro-pathologist (JW). The same pathologist had also assessed GS of biopsy in these men. The 30 patients recruited into the study were classified into low-, intermediate-, and high-risk categories according to Gleason grading that was undertaken according to standard diagnostic criteria. Low-risk cases were of GS 3 + 3 (*n* = 3), intermediate-risk GS cases were those with a GS of 3 + 4 (*n* = 13), and high-risk cases were of GS 4 + 3 and higher (*n* = 14). Representative blocks of the tumor were selected for subsequent immunohistochemical and gene expression studies. These men were followed by protocol-based interval PSA. Any biochemical recurrence (PSA > 0.2) was recorded. We also calculated upgrading/downgrading of GS from biopsies to radical surgery in this cohort

### 2.2 Ultrasound Shear Wave Elastography

Transrectal image acquisition for USWE was performed by using a sonographic push pulse to generate shear waves. A dynamic map of tissue stiffness (described as a Young’s Modulus) reflected as a different speed of shear waves in each tissue area in real time (29, 30) was obtained. Details of USWE have been described by us and others in previous publications (24, 31, 32). The prostate was subjected to the minimum possible pressure while remaining in contact with the probe for 5 to 10 s to ensure a stable acquisition of images. All USWE images were taken with a transrectal endocavitory transducer (SuperSonic imaging, Aix en Provence, France) with patients in lateral or lithotomy positions. USWE mode was used and elastograms of the prostate were acquired for each prostate lobe from cranial to caudal direction. Because the USWE field of view was insufficient to assess the whole prostate, the right and left lobes were scanned individually. The USWE images were obtained in transverse planes using a slow movement that allowed stabilization of the signals with gaps of 4 to 6 mm from base to apex. The most suspected lesions in planes were rescanned in 2- to 3-mm gaps. These suspicious locations were also inspected by rotating transducers in different directions to confirm abnormalities and estimate their sizes. The most suspect planes containing cancer were labeled and reconstructed offline into 3D images. Images (from red to blue for high to low stiffness, respectively) for each region were digitally saved, and the stiffness was quantitatively measured. The ratio between abnormal and normal regions was also measured. The images of the participants were then utilized to create specific prostate molds, which were used to guide prostate slicing postoperatively. A qualified urologist with more than 10 years of experience in transrectal ultrasound conducted the USWE. Based on data from the same study (31), we categorized tissue stiffness into <50, 50–100, 101–150, 151–200, 201–250, and 251–300. The person in charge of ultrasound and responsible for categorization was not aware of immunohistochemistry and gene expression results.

### 2.3 Immunohistochemistry

Sections from a representative block of the tumor were processed for immunohistochemical studies to identify CAFs. A panel of six antibodies (FAP, SMAα, FSP-1, CD36, PDGFR-α, and PDGFR-β) was utilized to screen tissue sections for CAFs in normal and neoplastic tissue. Antigen retrieval and de-paraffinization were performed using DAKO EnVision™ FLEX Target Retrieval solution (high pH) (Agilent Technologies) buffer in a DAKO PT Link. Sections were blocked in EnVision™ FLEX Peroxidase-Blocking reagent for 5 min at room temperature and incubated overnight at 4°C with anti-Alpha Smooth Muscle Actin (cat # NCL-L-SMA, Leica Biosystems) at a dilution of 1:50, anti-CD36 [C1C3] (cat no. GTX100642, GeneTex, Inc) at a dilution of 1:250, anti-FAP [JA56-11] (cat # MA5-32670, ThermoFisher Scientific) at a dilution of 1:100, anti-PDGFRα [D1E1] (#3174, Cell Signaling Technology) at a dilution of 1:100, anti-PDGFRβ [SY10-08] (cat #MA5-32047, ThermoFisher Scientific) at a dilution of 1:100, and S100A4 [EPR14639(2)] (cat # ab220213, abcam) at a dilution of 1:5,000. Immunostaining using DAKO EnVision™ FLEX system (Agilent Technologies) on a DAKO Autostainer Link48 was carried out according to the manufacturer’s protocol; more details are presented in **Table 1**. DAKO substrate working solution was used as a chromogenic agent for 2 × 5 min and sections were counterstained with EnVision™ FLEX hematoxylin. Sections known to stain positively were included in each batch and negative controls were prepared by replacing the primary antibody with DAKO antibody diluent. The “Quick” score method of assessment was used to determine the range of immunostaining performed. In this method, the intensity of the immunohistochemical reaction that was seen through the microscope was recorded as follows: 0, negative (no staining of any nuclei even at high magnification); 1, weak (only visible at high magnification); 2, moderate (visible at low magnification); and 3, strong (strikingly positive even at low magnification). The percentage of tumor nuclei that stained positively was also reported as 0 (none); 1 (approximately 1%–25%); 2 (26%–50%); 3 (51%–75%); or 4 (76%–100%). The intensity score was added to the proportion score to give the Quick score, which ranged from 0 to 7 for each tumor (33, 34).

**Table 1:** Antibody information and IHC conditions.

Antibody	Catalogue #	Company	Type	Tissue Control	Dilution
**CD36 (C1C3)**	GTX100642	Genetex	Poly Rabbit	Kidney	1/250
**FAP (JA56-11)**	MA5-32670	Invitrogen	Mono Rabbit	Testis	1/100
**FSP-1 (S100A4)**	Ab220213	Abcam	Mono Rabbit	Gastric Ca	1/5000
**PDGFRα (D1E1E)**	#3174	Cell Signaling	Mono Rabbit	GIST	1/100 + L
**PDGFRβ (SY10-08)**	MA5-32047	Invitrogen	Mono Rabbit	Spleen	1/100
**SMA Alpha**	NCL-L-SMA	Leica	Mono Mouse	Small bowelAppendix	1/50 + L

### 2.4 Gene Expression

In this study, we used TempO-Seq assays (BioSpyder., Inc. BIOCLAVIS, LTD, Biotechnology company in Glasgow, Scotland) to target sequencing-based RNA expression analysis on FFPE tissue sections of the human prostate. From each case, an area of 10 mm^2^ was dissected from normal and malignant prostate tissue and submitted for analysis. Each area was dissected using fresh blades to avoid cross-contamination of the tissue samples. The samples were collected into sterile Eppendorf tubes and were then analyzed using appropriate internal process controls (BioClavis) as well as with the Human Whole Transcriptome v2.0 panel with standard attenuators.

#### 2.4.1 TempO-Seq Assay Protocol Summary

**Figure 2:**
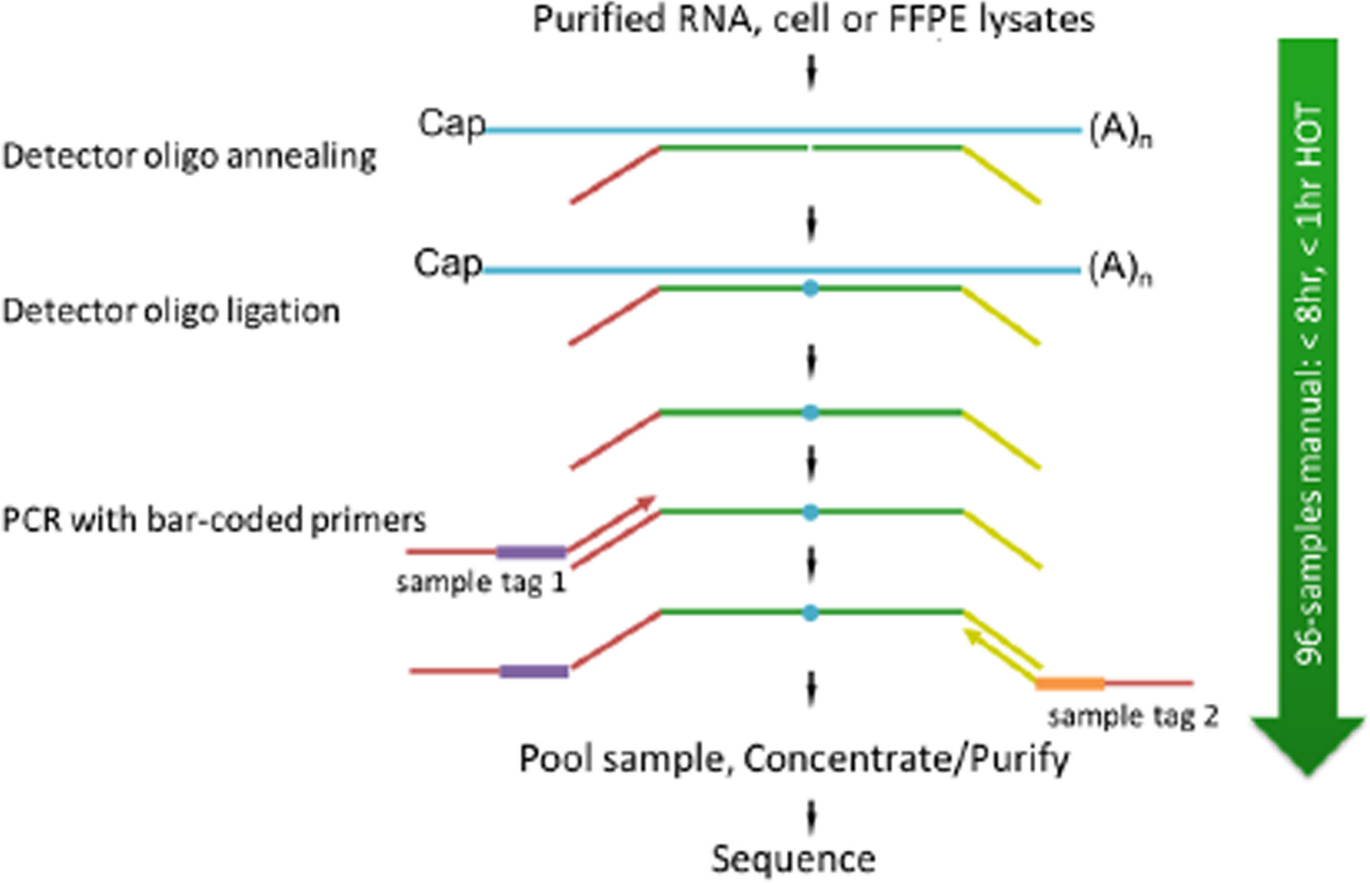
Schematic of TempO-Seq assay protocol.

Sequencing libraries for targeted panels were generated as illustrated in **Figure 2**.

In TempO-Seq, each Detector Oligo comprises a sequence complementary to an mRNA target plus a universal (i.e., same for every targeted gene) primer binding site. They anneal in immediate juxtaposition to each other on the targeted RNA template such that they can be ligated together. Then, the ligated detector oligos are PCR-amplified using a primer set (single-plex PCR reaction, with a single primer pair for each sample) that introduces both the adaptors required for sequencing and a sample-specific barcode. The barcode sequences flank the target sequence and are appropriately inserted into the standard Illumina adaptors to permit standard dual-index sequencing of the barcodes and deconvolution of sample-specific reads from the sequencing data using the standard Illumina software. All the PCR-amplified and barcoded samples are combined into a single library for sequencing. Sequencing reads are de-multiplexed using the standard sequencing instrument software for each sample using the barcodes to give a FASTQ file for each. The genes of interest, which are PDGFRα, PDGFRβ, CD36, FSP1, FAP, and SMAα, were identified for further analysis.

### 2.5 Data Analysis

SPSS v.22 (SPSS, Chicago, IL, USA) was used for all statistical analyses. The following data were collected: Patient’s age (in years), prostate-specific antigen (PSA, in ng/ml), prostate volume (in ml), modulus measurement using USWE (in kPa), Gleason score (GS), and immunostaining “Quick” score (in a scale of 0-7) as well as gene expression for the six genes of interest (PDGFRα, PDGFRβ, CD36, FSP1, FAP, and SMAα). Continuous data were first tested to see if they were normally distributed by the Kolmogorov–Smirnov Test of Normality. The mean (m) and standard deviation (SD) were described if the variable followed a normal distribution. The median (M) and interquartile range (IQR) were presented if the variable did not follow a normal distribution. In this study, a GS ≥ 4 + 3 was considered to be significant PCa following the UCL 2 definition. A Spearman rank correlation was conducted to examine the relationship between GS of radical prostatectomy and tissue stiffness (in kPa) using USWE. Correlations between cancer and normal stroma of IHC and gene expression were analyzed using Spearman’s rank correlation and Pearson correlation. A *p*-value of 0.01 was considered to be statistically significant in correlation procedures.

## 3 Results

The clinicopathological data of the cohort are presented in **Table 2**. Thirty patients with a mean age of 67.8 (SD, 5.4) years were treated by radical prostatectomy with a median PSA level of 9.7 (IQR, 17.5–7.9) ng/ml and a mean prostate volume of 64.2 (SD, 32.9) ml.

**Table 2:** Patient and imaging characteristics.

Patient characteristic	
**No. Pts**	30
**Age (in years)**	
Mean ± SD	67.8 ± 5.4
**PSA level (ng/ml)**	
Median (IQR)	9.7 (17.5–7.9)
**Prostate volume**	
Mean ± SD	64.2 ± 32.9
**Stiffness measurement using USWE in kilopascals (mean ± SD)**	**No. (%)**
<50	0 (0.0)
50–100 (92.1 ± 5.4)	4 (13.3)
101–150 (121.3 ± 11.6)	20 (66.7)
151–200 (170.3 ± 9.9)	5 (16.7)
201-250	0 (0)
251–300	1 (3.3)
**Gleason Score**	**No.(%)**
3+3	3 (10.0)
3+4	13 (43.3)
4+3	5 (16.7)
3+5	4 (13.3)
4+5 or greater	5 (16.7)

In this study, there was a positive correlation between USWE-measured tissue stiffness and GS in medium strength, which is statistically significantly measured by Spearman rank correlation (*r*_s_ = 0.67, *p* < 0.001, df = 28). Tissue stiffness increased with GS.

### 3.1 Correlation Between Tissue Stiffness and Immunohistochemistry

Representative whole slides from each of the radical prostatectomy specimens were stained using the six antibodies to detect the CAFs and the Quick score method was used for scoring the staining intensity in normal and cancer tissue.

**Figure 3:**
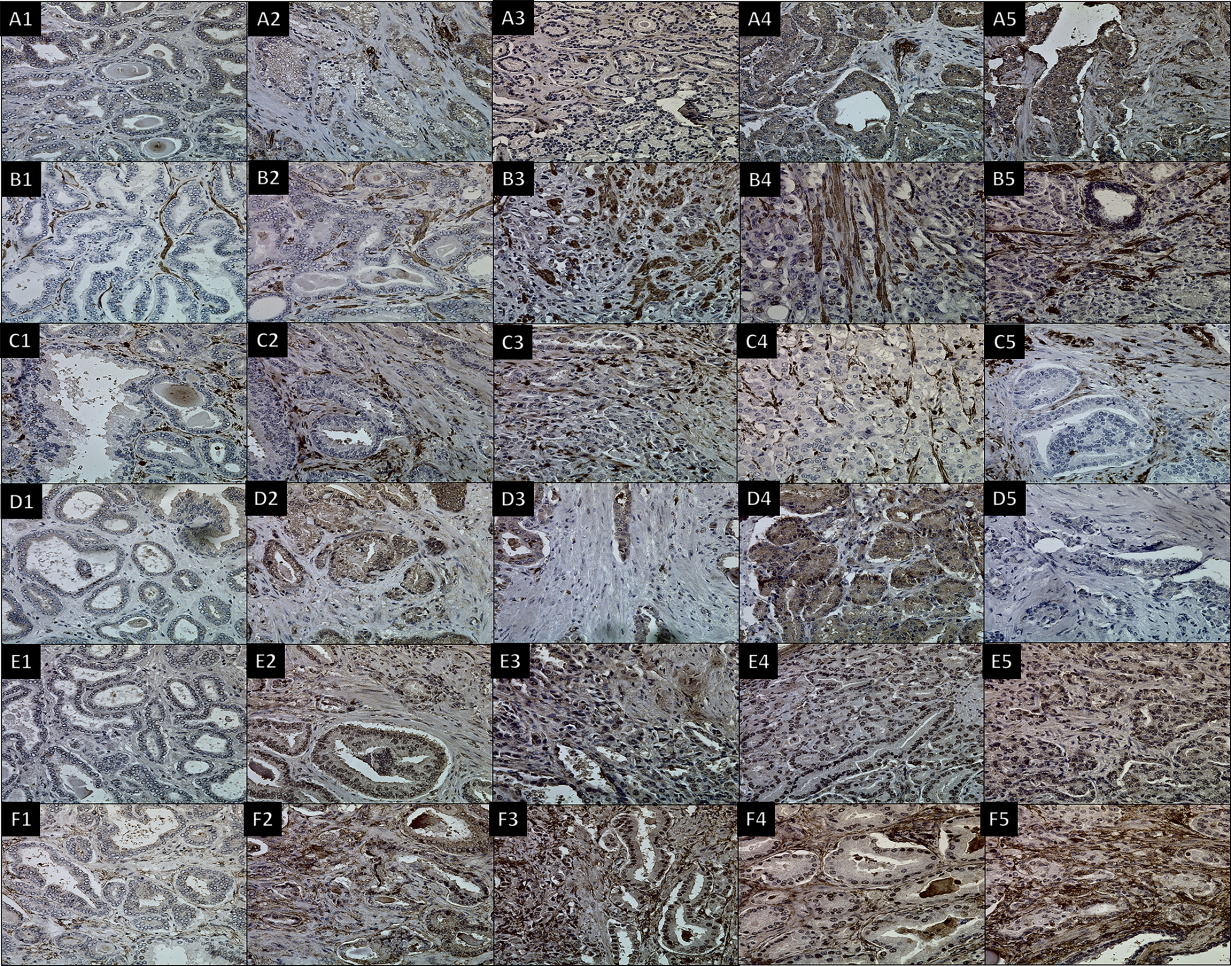
IHC using six antibodies to stain CAFs in different Gleason scores of prostate cancer tissues. Images for **(A)** FAP with dilution (1/100), **(B)** SMAα with dilution (1/50 + linker), **(C)** FSP1 with dilution (1/5,000), **(D)** CD36 with dilution (1/250), **(E)** PDGFRα with dilution (1/100 + linker), and **(F)** PDGFRβ with dilution (1/100). (1) 3 + 3 GS, (2) 3 + 4 GS, (3) 4 + 3 GS, (4) 3 + 5 GS, and (5) 4 + 5 GS. 400× magnification.

**Figure 4:**
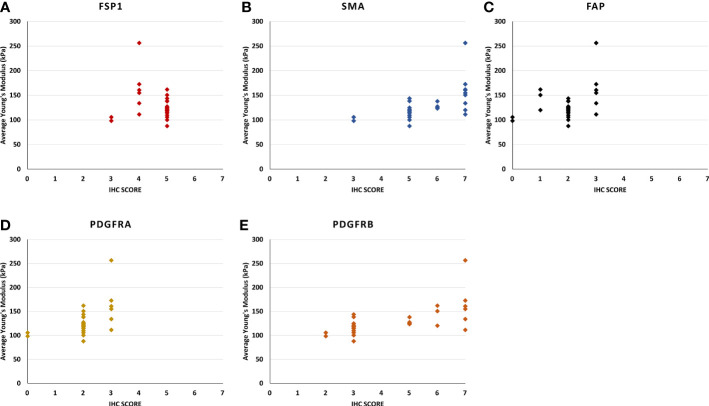
The scatter plots show the relationship between the stiffness of the Ultrasound shear wave elastography and the score of immunohistochemistry intensity and density in **(A)** FSP1, **(B)** SMAα, **(C)** FAP, **(D)** PDGFRα, and **(E)** PDGFRβ. **(B)** [rs (28) = 0.73, p < 0.0010], and **(E)** [rs (28) = 0.73, p < 0.001], the correlation of the other genes were not statistically significant.

For FAP and PDGFRα, weak staining was observed in the fibromuscular stroma compared to the positively stained regions in the tumor epithelium (**Figures 3A-E**). The correlation between increasing the tissue stiffness and expression of FAP and PDGFRα in the fibromuscular stroma was not significant as presented in **Figures 4C, D**.

For SMAα, the intensity and density of staining were scored. Strong staining was observed in the fibromuscular stroma compared to the negatively stained in normal epithelium. SMAα was expressed at a higher level in the high-grade cancers [GS (4 + 5)] compared to the lesser grade PCa as shown in **Figure 3B**. There was a significant strong positive correlation between tissue stiffness and SMAα expression in terms of IHC score in the fibromuscular stroma [*r*_s_(28) = 0.909, *p* < 0.001] as presented in **Figure 4B**. Also, PDGFRβ was strongly expressed in the fibromuscular stroma and was expressed at a higher level in the high-grade PCa compared to lower grade PCa similar to SMAα as presented in **Figure 3F**. Similar to SMAα, there was a significant strong positive correlation between increasing tissue stiffness and PDGFRβ expression in terms of IHC score in the fibromuscular stroma [*r*_s_(28) = 0.925, *p* < 0.001] as shown in **Figure 4E**. The Spearman rank correlation results also showed a significant strong positive correlation between GS increase and the increase of SMAα [*r*_s_(28) = 0.912, *p* < 0.001] and PDGFRβ [*r*_s_(28) = 0.925, *p* < 0.001] expression in terms of IHC score, respectively.

For FSP1, the intensity of staining was scored high in tumor fibromuscular stroma compared to the negatively stained epithelium. When comparing intermediate-grade PCa to low- and high-grade PCa, strong staining was observed in the cancerous fibromuscular stroma as shown in **Figure 3C**. There was no significant correlation between increasing the tissue stiffness and expression of FSP1 in the fibromuscular stroma as shown in **Figure 4A**.

For CD36, there was no expression of staining in both tumor and fibromuscular stroma compared to the positively stained normal epithelium as presented in **Figure 3D**. **Table 3** shows the evaluation and calculation of the average pixel intensity and the percentage of the positive staining pattern of the IHC images of the six antibodies using ImageJ.

**Table 3:** Using ImageJ to evaluate and calculate the average pixel intensity and the percentage of the positive staining pattern of the IHC images.

Antibody	Gleason score	Area	Staining area %	The mean of staining area	Location of staining (nuclei or fibromuscular stroma)
FAP	3+3	4194304	9.8	7.9	Nuclei
	3+4	4194304	2.1	17.5	Nuclei
	4+3	4194304	4.5	18.3	Nuclei
	3+5	4194304	15.7	44.5	Nuclei
	4+5	4194304	15.4	56.0	Nuclei
SMA**α**	3+3	4194304	5.1	9.2	Fibromuscular stroma
	3+4	4194304	7.7	19.4	Fibromuscular stroma
	4+3	4194304	16.7	35.3	Fibromuscular stroma
	3+5	4194304	21.7	40.1	Fibromuscular stroma
	4+5	4194304	25.7	45.1	Fibromuscular stroma
FSP1	3+3	4194304	6.9	6.6	Fibromuscular stroma
	3+4	4194304	8.8	8.4	Fibromuscular stroma
	4+3	4194304	8.7	16.9	Fibromuscular stroma
	3+5	4194304	9.6	25.4	Fibromuscular stroma
	4+5	4194304	9.7	21.9	Fibromuscular stroma
CD36	3+3	4194304	0.7	2.4	Nuclei
	3+4	4194304	2.8	18.4	Nuclei
	4+3	4194304	2.2	7.5	Nuclei
	3+5	4194304	14.6	35.2	Nuclei
	4+5	4194304	0.7	0.8	Nuclei
PDGFRα	3+3	4194304	0.5	2.6	Nuclei
	3+4	4194304	7.9	26.4	Both
	4+3	4194304	14.3	26.6	Both
	3+5	4194304	11.5	44.1	Both
	4+5	4194304	12.5	44.7	Both
PDGFRβ	3+3	4194304	1.4	3.9	Fibromuscular stroma
	3+4	4194304	8.3	30.3	Fibromuscular stroma
	4+3	4194304	11.3	39.3	Fibromuscular stroma
	3+5	4194304	15.8	59.0	Fibromuscular stroma
	4+5	4194304	25.4	96.5	Fibromuscular stroma

### 3.2 Correlation Between Tissue Stiffness and Gene Expression

**Figure 5:**
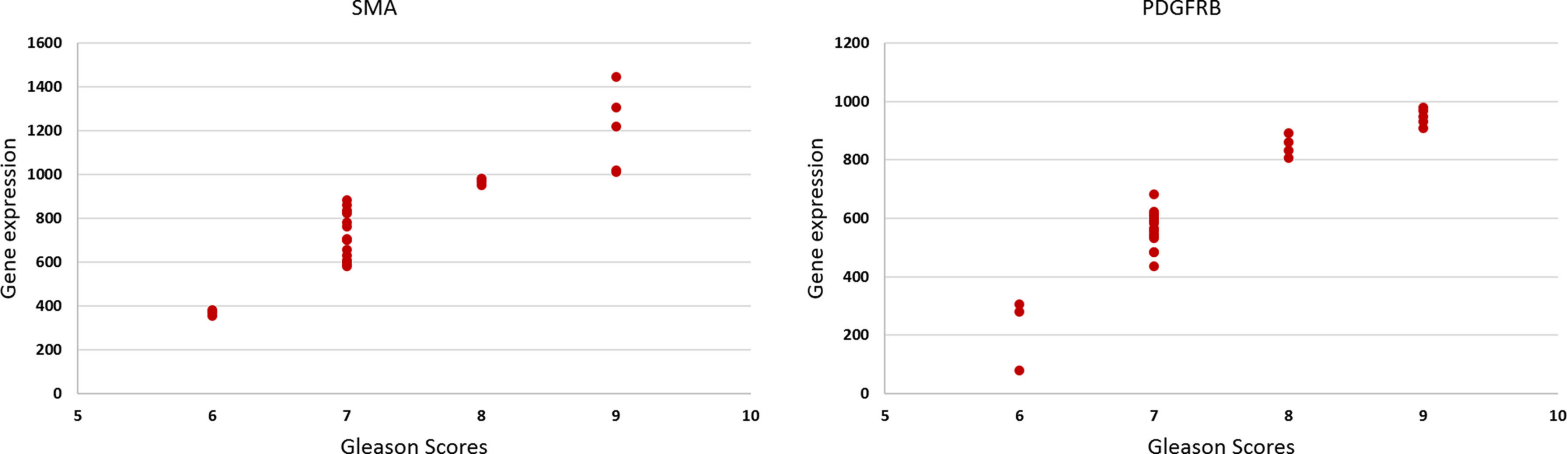
The scatter plots show the relationship between the Gleason score and the gene expression of the SMAα and PDGFRβ [*r*_s_(28) = 0.58, *p* = 0.001, and *r*_s_(28) = 0.56, *p* = 0.001], respectively.

The results of the gene expression for PDGFRα showed a bimodal pattern. The expression was low in GS 3 + 3 and 4 + 5 cancer stroma compared to the normal stroma. However, for 3 + 5, 3 + 4, and 4 + 3 GS cancers, the gene expression is higher in cancer stroma than normal stroma. FSP1 and FAP have little expression in both normal and cancer stroma in all GS cancers. CD36 has no expression in all GS. Conversely, SMAα and PDGFRβ have the best gene expression results supporting the IHC results, and the gene expression increased gradually with an increase in the GS as presented in **Figure 5**. There was a significant positive correlation between SMAα and PDGFRβ gene expression in the fibromuscular stroma and GS [*r*_s_(28) = 0.882, *p* < 0.001, and *r*_s_(28) = 0.881, *p* < 0.001, respectively]. When evaluating the relationship between gene expression of SMAα and PDGFRβ and tissue stiffness in the tumor stroma, Pearson correlation results showed a significant positive correlation between SMAα [*r*(28) = 0.706, *p* < 0.001] and PDGFRβ [*r*(28) = 0.809, *p* < 0.001] gene expression in the fibromuscular stroma and increasing tissue stiffness.

## 4 Discussion

The present study explored the mechanistic and immunohistochemical features of the extracellular matrix of PCa of varying GSs in comparison to normal benign prostate tissue. The results were validated using a novel TempO-sequence assay gene expression methodology. We have focused on both quantitative imaging of the tumors and their microenvironment, thus providing a comprehensive insight into the PCa disease microenvironment.

In a heterogeneous disease such as PCa, research needs to focus on understanding cellular architecture including TME in order to accurately predict the behavior of cancer including the development of metastatic disease. The study used different modes of analysis, including genomic sequencing, USWE, and IHC to characterize tumor heterogeneity and TME. A comprehensive tumor characterization can lead to an increased understanding of the risk stratification of PCa for precise diagnosis and treatment. TME characterization using three different modalities improved our ability to predict more aggressive cancers and their outcomes. There have been no investigations to date of tissue-level spatial heterogeneity in CAFs in PCa TME and how they relate to the radiological appearance of these tumors on USWE-measured quantitative tissue stiffness. Multiscale characterization, such as that carried out in the present study, provides identification of quantitative metrics derived from non-invasive imaging, i.e., USWE, which correlate with or predict aggressive PCa phenotypes. These metrics may be made available to physicians making decisions for new patients with biopsy confirmed PCa to choose the best therapeutic option based on the presence of certain TME characteristics that may affect treatment response or outcome.

The multimodal and multiscale investigative workflow used in our study provides a new option for RNA extraction of formalin-fixed tissues using a TempO-sequence platform in PCa for next-generation sequencing and genomic analysis. Again, to our knowledge, this has not been reported. The technique may be useful to get information from archived tissues with long-term follow-up of patient cohorts.

**Figure 6:**
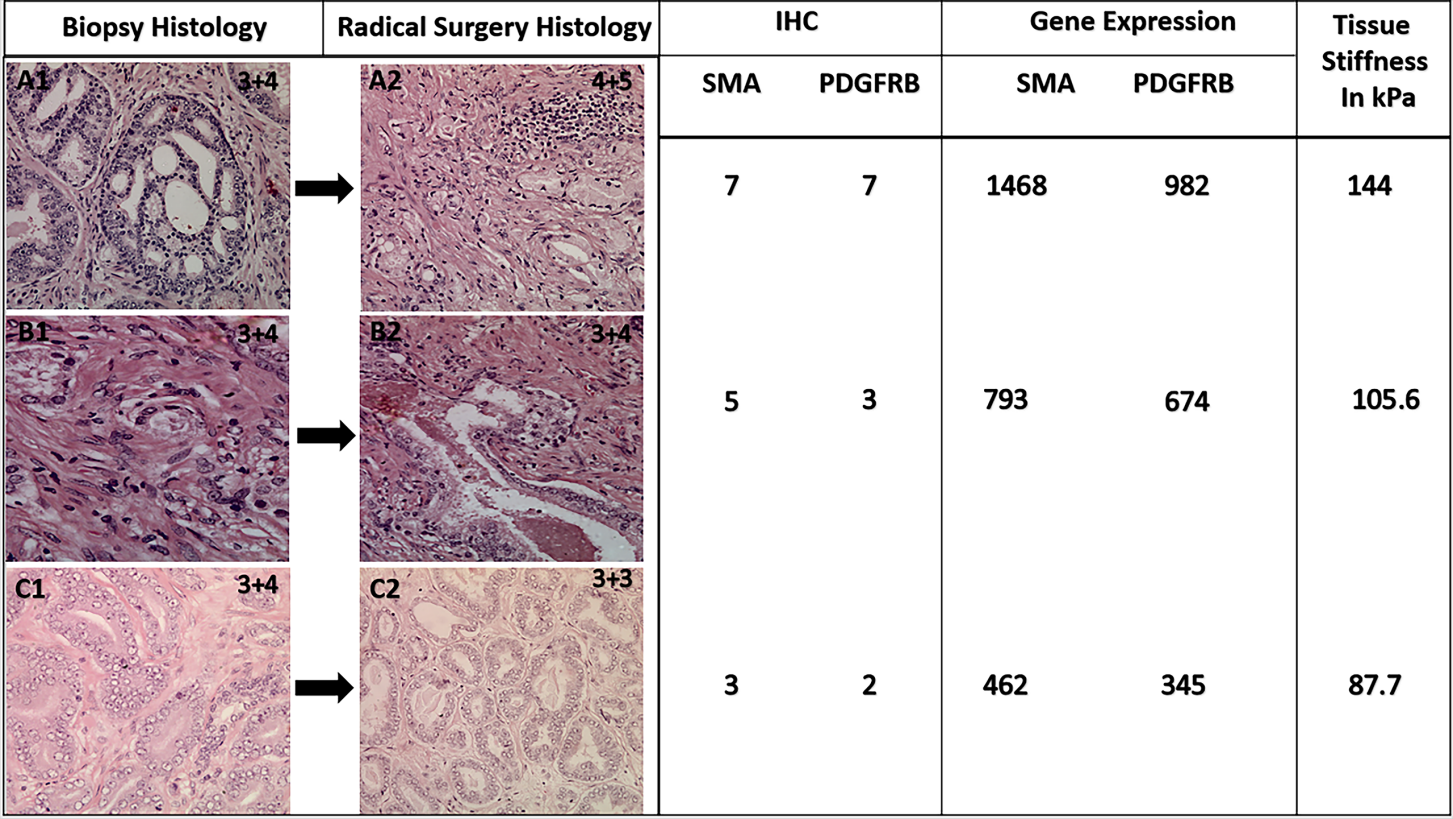
Representative figure showing a change in histological GS following radical surgery and corresponding expression of CAFs and genomic analysis. Note tissue stiffness measured in kPa using ultrasound shear wave elastography.

We observed a consistent and coherent pattern of increased GS correlating with increased tissue stiffness on USWE and increased expression of SMAα and PDGFRβ both on IHC and genomics analysis. The workflow also suggested that genomics expression for SMAα and PDGFRβ along with measurement of stiffness using USWE could narrow the discrepancy gap between histopathology of biopsies and radical surgery (**Figure 6**). A better prediction of aggressive cancer phenotype would improve risk stratification and treatment strategies. Use of biomarkers, both genomics and immunohistochemistry, to risk stratify PCa would require validation of our results in a larger cohort of men using multicenter data. We envisage that the findings of the study would act as a driver to change clinical practice, especially in men where biopsy has under-sampled PCa and/or does not correlate with the rise in PSA level or the findings of multi-parametric MRI.

In this study, a panel of six antibodies showed some staining heterogeneity in the fibromuscular stroma. However, some of those markers were difficult to interpret, but PDGFRβ and SMAα are the two antibodies that gave crisp and strong staining of the CAFs and the intensity of the staining significantly increased with increasing the USWE-measured stiffness and GS of PCa. In corroboration of our findings, Yngve et al. found the mean expression of PDGFRβ to be higher in tumor stroma compared to the normal stroma (35). Masko et al. found that the metastasis breast stroma has higher SMAα expression compared to the normal stroma (36). According to some studies, higher SMAα expression in stromal cells correlates with tumor aggressiveness, progression, and worse prognosis (37, 38). To confirm the IHC result, a gene expression was carried out for the six antibodies. The results validated and supported the results of the IHC of the SMAα and PDGFRβ.

A comprehensive illustration of TME and measurement of stiffness may allow us to understand mechanosensing, which influences directional cell migration. The mechanosensing mechanisms of cancer cells change the growth factor signalling of cells including secretion of proteolytic enzymes, which degrade the fiber matrix of cancers. Some of these changes may explain enhanced growth, survival, and invasion/metastases of tumors. Increased tumor tissue stiffness has been associated with a higher risk of biochemical recurrences following radical treatment of localized PCa (32). Previous studies have linked tumor tissue stiffness to cancer progression, stem cell differentiation, and expression of genes involved in invasion and metastasis; thus, mechanistic insights and CAF heterogeneity from our study might explain part of this (39, 40). Interestingly, Nordby et al. have reported high expression of PDGFRβ to be an independent prognostic marker on multivariate regression analysis for clinical and biochemical recurrence of men following treatment for localized disease (35).

There are several clinical implications of the present study. The findings should make us look at cancer as a disorder in the local microenvironment rather than a disease confined to cells alone. A positive correlation between CAF heterogeneity and stiffness on USWE provides us a non-invasive way to image the cancer microenvironment. This opens us to a new area that has prognostic and therapy monitoring prospects in novel treatments such as extracorporeal shock waves (41). PCa is a clinically and molecularly heterogeneous disease and we need better prognostic biomarkers for risk stratification of patients for various treatment options.

The second use of these findings could help us in narrowing the gap of GS upgrading between biopsies and radical surgery. GS of PCa on diagnostic biopsies is known to get upgraded following radical prostatectomy histopathology. The upgrade rate of biopsy GS on radical prostatectomy is about 35.5% (range: 14%–51%) (42). This remains a crucial challenge to overcome, in particular when this is utilized for risk assessment in men with a recent PCa diagnosis (43). We found increased expression of SMAα and PDGFRβ in ECM around tumors correlated with GS in radical prostatectomy histopathology in our study. Whether expression of these markers in biopsies would predict upgrading of GS needs further study; however, our previously reported results indicated a significant advantage of using USWE as predicting and detecting technique for PCa grade (32), and it would impact the choice of therapeutic approach and management of PCa.

There were some limitations in this study. First, we did not investigate the organization of the collagen types that were produced by CAFs and the disorder of the fibers in the tumors. However, this study aimed to distinguish the different elasticities of PCa investigating the causes of stiffness in USWE using IHC. In a recently published study (44), we have investigated the collagen types and orientation in causing tissue cancer stiffness. Another limitation is that this study is from a single institution with an experienced operator performing the test, most certainly USWE.

Findings from the present study should add to further knowledge on the mechanism of PCa disease. This will also add and open future avenues for designing PCa therapeutics and interventions including focal therapy.

## Conclusion

USWE-measured tissue stiffness correlates with the degree of CAF heterogeneity of TME on the immunohistochemical characterization of localized PCa. Expression of SMAα and PDGFRβ is strong in TME and correlates with USWE tissue stiffness.

## Data Availability Statement

The original contributions presented in the study are included in the article/supplementary materials. Further inquiries can be directed to the corresponding author.

## Ethics Statement

The studies involving human participants were reviewed and approved by East of Scotland ethics committee. The patients/participants provided their written informed consent to participate in this study.

## Author Contributions

Conceptualization: GN and CL Methodology: WA, XZ, CO, SB, NK, JW, CL and GN. Investigation: WA, XZ, CO, SB, NK, JW, CL, and GN. Writing—original draft: WA and GN. Reviewing and editing: WA, XZ, CO, SB, NK, JW, CL, and GN. Supervision: GN and CL. All authors have read and agreed to the published version of the manuscript.

## Conflict of Interest

The authors declare that the research was conducted in the absence of any commercial or financial relationships that could be construed as a potential conflict of interest.

## Publisher’s Note

All claims expressed in this article are solely those of the authors and do not necessarily represent those of their affiliated organizations, or those of the publisher, the editors and the reviewers. Any product that may be evaluated in this article, or claim that may be made by its manufacturer, is not guaranteed or endorsed by the publisher.
